# Downregulation of CDC27 inhibits the proliferation of colorectal cancer cells via the accumulation of p21^Cip1/Waf1^

**DOI:** 10.1038/cddis.2015.402

**Published:** 2016-01-28

**Authors:** L Qiu, J Wu, C Pan, X Tan, J Lin, R Liu, S Chen, R Geng, W Huang

**Affiliations:** 1 Sun Yat-sen University Cancer Center, State Key Laboratory of Oncology in South China, Collaborative Innovation Center for Cancer Medicine, Guangzhou, China; 2 Medical Oncology, Sichuan Cancer Hospital and Institute, Second People's Hospital of Sichuan Province, Chengdu, China

## Abstract

Dysregulated cell cycle progression has a critical role in tumorigenesis. Cell division cycle 27 (CDC27) is a core subunit of the anaphase-promoting complex/cyclosome, although the specific role of CDC27 in cancer remains unknown. In our study, we explored the biological and clinical significance of CDC27 in colorectal cancer (CRC) growth and progression and investigated the underlying molecular mechanisms. Results showed that CDC27 expression is significantly correlated with tumor progression and poor patient survival. Functional assays demonstrated that overexpression of CDC27 promoted proliferation in DLD1 cells, whereas knockdown of CDC27 in HCT116 cells inhibited proliferation both *in vitro* and *in vivo*. Further mechanistic investigation showed that CDC27 downregulation resulted in G1/S phase transition arrest via the significant accumulation of p21 in HCT116 cells, and the upregulation of CDC27 promoted G1/S phase transition via the attenuation of p21 in DLD1 cells. Furthermore, we also demonstrated that CDC27 regulated inhibitor of DNA binding 1 (ID1) protein expression in DLD1 and HCT116 cells, and rescue assays revealed that CDC27 regulated p21 expression through modulating ID1 expression. Taken together, our results indicate that CDC27 contributes to CRC cell proliferation via the modulation of ID1-mediated p21 regulation, which offers a novel approach to the inhibition of tumor growth. Indeed, these findings provide new perspectives for the future study of CDC27 as a target for CRC treatment.

Despite the increased understanding of its pathogenic risk and the development of progressive therapeutic strategies, colorectal cancer (CRC) remains a major cause of cancer morbidity and ranks as the second leading cause of cancer death in China.^1, 2, 3, 4^ CRC development is a multistep process that involves complex cascades of molecular events,^5^ therefore understanding the molecular mechanisms that drive malignant tumor formation and progression will contribute to the identification of novel molecular targets and provide more effective methods for CRC prevention and therapy.

The anaphase-promoting complex/cyclosome (APC/C) is a major regulator of protein degradation during mitosis,^6, 7, 8^ consists of at least 14 subunits^9^ and are involved in various critical cellular events, such as mitotic progression, and controlling differentiation.^10, 11^ APC/C degrades its substrates through binding to two co-activators: CDH1 and cell division cycle 20 (CDC20). CDC20 is active in early mitosis, whereas CDH1 activity is restricted to late mitosis and the G1 phase.^12^ Cell division cycle 27 (CDC27) is a core subunit of APC/C that is responsible for binding CDH1 and CDC20, thereby activating APC/C to recognize and degrade target substrates.^13^ Emerging evidence has revealed that CDC20 and CDH1 have opposing functions in tumorigenesis. CDH1 has been shown to function largely as a tumor suppressor, whereas CDC20 exhibits an oncogenic function, which was observed in a variety of human tumors.^14, 15, 16, 17, 18^ However, despite being a key binding partner of CDC20 and CDH1, there is no research on whether CDC27 has a role in cancer. However, recent sequencing studies have reported that *CDC27* mutation is detected in various cancers.^19, 20, 21, 22^ In particular, for CRC, the *CDC27* mutation rate was more than 5%, which attracted our interest in exploring the potential function of CDC27 in CRC formation and progression.^23^

Inhibitor of DNA binding 1 (ID1) is a negative regulator of basic helix–loop–helix transcription factors and is involved in regulating a variety of cellular processes, including growth, senescence, and differentiation.^24, 25, 26, 27, 28^ The overexpression of ID1 has been observed in a variety of cancers where it contributes to tumor formation and invasion.^26^ Previous studies have demonstrated that ID1 transcriptionally inhibits the expression of the cyclin-dependent kinase inhibitors p21 in prostate cancer.^29, 30^ Nevertheless, the relationship between CDC27 and ID1 in cancer cells remains unknown.

In the present study, we aimed to explore whether CDC27 has a key role in CRC, and our findings provide new insights into the mechanisms of CRC tumorigenesis and support the potential of CDC27 as a therapeutic target in CRC treatment.

## Results

### CDC27 is frequently upregulated in CRC cell lines and tumors

**Figure 1:**
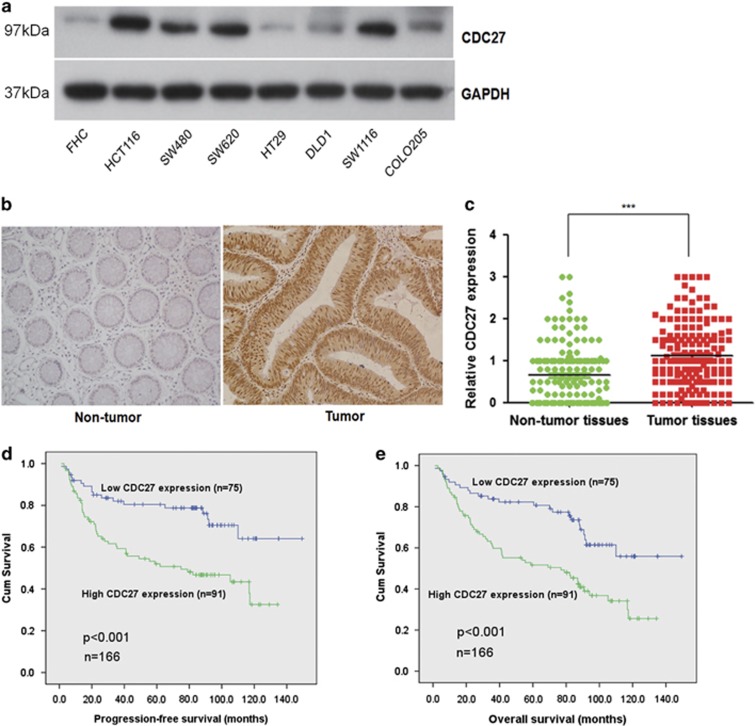
CDC27 is overexpressed in CRC cell lines and patient tissues and is associated with decreased survival in CRC. (**a**) Upregulation of CDC27 protein expression was detected by western blotting analysis in a normal colon epithelial cell line (FHC) and seven CRC cell lines. GAPDH was used as a reference control. (**b**) Representative images of CDC27 expression in CRC tissues and paired adjacent normal mucosal tissues by immunohistochemistry (× 200 magnification). (**c**) Relative immunohistochemstry analysis for CDC27 expression in CRC tissues and adjacent normal mucosal tissues; each point in the graph represents the CDC27 expression H-score of an individual patient tumor (*n*=166, ***P<0.001, paired Student's *t*-test). (**d** and **e**) Kaplan–Meier survival analysis of the association between CDC27 expression and PFS or OS (log-rank test)

To investigate the role of CDC27 in human CRC development, we first examined CDC27 expression at the protein level in eight CRC cell lines using western blotting. Western blotting analysis revealed that CDC27 protein expression was upregulated in CRC cell lines compared to the normal colon epithelial cell line FHC (Figure 1a). Furthermore, we evaluated endogenous CDC27 expression by immunohistochemistry using paraffin-embedded tissue sections (*n*=166) of histopathologically confirmed CRC. This analysis revealed that CDC27 was predominantly expressed in the nucleus, and tumor tissues showed a stronger staining intensity compared with paired adjacent non-tumor tissues (Figures 1b and c).

### CDC27 expression is positively correlated with cancer progression and proliferation of CRC

To understand the functional significance of CDC27 in CRC, we analyzed the relationship between CDC27 expression and the clinicopathological features of CRC patients. The clinical characteristics of the patients are summarized in Table 1. High positive CDC27 expression was detected in 91 (54.8%) of the tumor tissue samples. Moreover, the results revealed that CDC27 expression was significantly associated with tumor size (*P*=0.035), TNM stage (*P*=0.013), and distant metastasis (*P*=0.03). Collectively, our results provide evidence that CDC27 is associated with CRC proliferation and progression.

### CDC27 overexpression is associated with decreased survival in CRC

Next, we analyzed the relationship between CDC27 expression and patient survival. The survival time of the 166 CRC patients ranged from 1.1 to 149.4 months. As shown in Figures 1d and e, the results showed that CDC27 expression in CRC was negatively correlated with patient progression-free survival (PFS) and overall survival (OS). Kaplan–Meier survival analysis and log-rank tests revealed that CDC27 expression was negatively associated with PFS (*P*<0.001, HR=2.518, 95% CI=1.481–4.281) and OS (*P*<0.001, HR=2.348, 95% CI=1.454–3.791). The rates of PFS (46.2% *versus* 74.7%) and OS (38.5% *versus* 68.0%) were significantly lower in the CDC27 high-expression group than that in the low-expression group. Multivariate Cox proportional hazards regression analysis indicated that CDC27 expression served as an independent prognostic factor for PFS (*P*=0.04, HR=1.78, 95% CI=1.03–3.07) and OS (*P*=0.02, HR=1.79, 95% CI=1.09–2.94; Table 2). Collectively, this analysis strongly indicates that CDC27 expression may serve as a potential independent prognostic factor for PFS and OS in CRC patients.

### Downregulation of CDC27 inhibits cell growth in colon cancer cells

**Figure 2:**
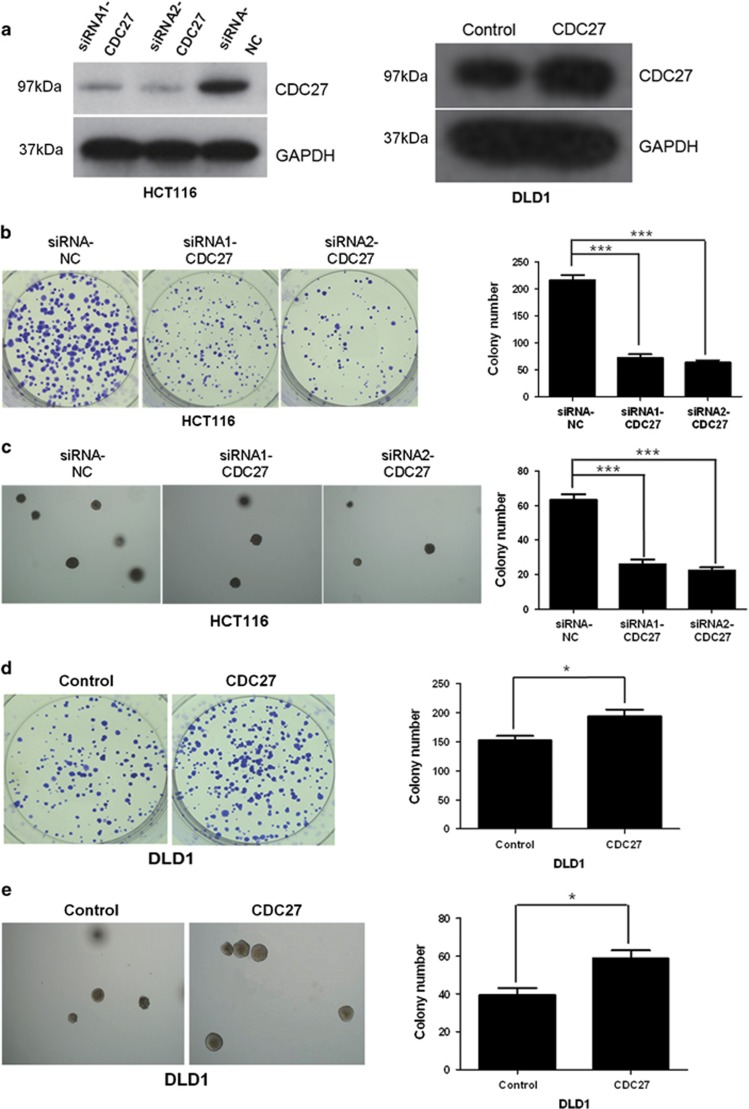
CDC27 promotes proliferation of CRC cells. (**a**) The efficient overexpression or suppression of CDC27 was identified by western blotting. (**b**) Representative images show the colony formation ability of HCT116 cells with CDC27 transiently suppressed. The number of the colonies was quantified. ***P<0.001 using Student's *t*-test. (**c**) Soft agar colony formation assays. Representative images are shown; the numbers of colonies containing more than 50 cells were scored. ***P<0.001 using Student's *t*-test. (**d** and **e**) CDC27 overexpression promoted cell growth in both colony formation assays and soft agar colony formation assays. Representative images are presented. *P<0.05 using Student's *t*-test. These experiments were repeated at least three times. Error bars, mean±S.D.

We next sought to investigate whether downregulation of CDC27 could affect the proliferation of CRC cells. HCT116 cells were transiently transfected with small interfering RNAs (siRNAs) targeting CDC27 (siRNA1-CDC27, siRNA2-CDC27) or negative control siRNA (siRNA-NC), and DLD1 cells were transiently transfected with CDC27-carrying plasmids or empty vector, respectively. The expression level of CDC27 in these cells was confirmed by western blotting (Figure 2a). The results of the colony formation assays indicated that the cells transfected with siRNA exhibited a weakened capacity for colony formation compared with the control group in HCT116 (Figure 2b). Similar results were obtained in another CRC cell line, SW480 (Supplementary Figure 1). We also performed soft agar colony formation assays, and the colony number of the CDC27 siRNA group was distinctly less than that of the control group in HCT116 cell line (Figure 2c). Moreover, exogenous CDC27 expression in DLD1 can promote cell proliferation (Figures 2d and e). Therefore, our results provide evidence that CDC27 can promote proliferation in CRC cell lines.

### CDC27 influences the G1/S phase transition of the cell cycle through regulating p21 expression

**Figure 3:**
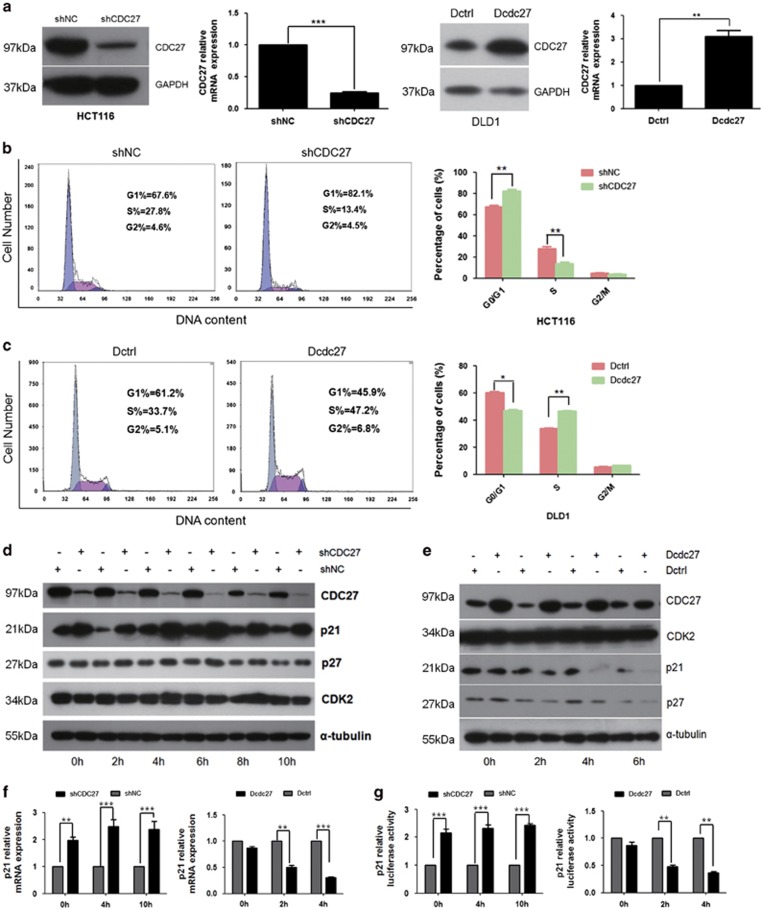
CDC27 inhibits p21-dependent arrest at the G1/S phase transition. (**a**) CDC27 overexpression in DLD1 stable cell lines and persistent suppression in HCT116 stable cell lines identified using real-time PCR and western blotting. Mean±S.D. of triplicate samples are shown. **P<0.01, ***P<0.001, Student's *t*-test. (**b** and **c**) The indicated stable cell lines were subjected to cell cycle distribution analysis by flow cytometry. Images and qualification of the cell cycle distribution in three independent experiments are shown; *P<0.05, **P<0.01, Student's *t*-test. (**d** and **e**) The HCT116 stable cell lines or DLD1 stable cell lines were synchronized by serum starvation for 24 h, and protein was harvested every 2 h after serum restoration. Western blotting analysis was used to detect changes in p21, p27 expression in the indicated cell lines. α-Tubulin was used as a reference control. (**f**) Cells were harvested at indicated time points after synchronization. Expression of p21 mRNA was analyzed by real-time PCR. Mean±S.D. of triplicate samples are shown. **P<0.01, ***P<0.001, Student's *t*-test. (**g**) pGL3-p21 was transfected into both HCT116 and DLD1 stable cell lines, and cells were harvested at the indicated time after synchronization for luciferase reporter assays. Mean±S.D. of triplicate samples are shown. **P<0.01, ***P<0.001, Student's *t*-test

Given our findings thus far, we conducted a cell cycle analysis to identify the mechanisms by which CDC27 downregulation can inhibit proliferation. To persistently suppress CDC27 expression, we constructed two HCT116 stable cell lines: shCDC27 and shNC. In addition, we constructed a stable DLD1 cell line overexpressing CDC27 (Dcdc27) and a control DLD1 cell line (Dctrl) to detect alterations in cell cycle distribution. The overexpression or knockdown efficiency was confirmed through real-time PCR and western blotting (Figure 3a). As shown in Figure 3b, HCT116-shCDC27 remarkably increased the number of cells in G1 phase compared with the control group. Correspondingly, overexpression of CDC27 promoted the G1/S transition in DLD1 stable cell lines (Figure 3c). To clarify the specific role of CDC27 in the G1/S phase transition, cells were synchronized at the G0/G1 phase and harvested every 2 h after recovery of the serum supply. Key regulators of the G1 phase were evaluated by western blotting. Intriguingly, we found that knockdown of CDC27 led to a dramatic accumulation of p21 protein at each time point examined, whereas p27 expression was only weakly increased at certain time points (Figure 3d). In addition, the expression of p21 remarkably decreased in the Dcdc27 cell lines at 2, 4, and 6 h after serum restoration compared with that in the Dctrl stable cell lines, whereas p27 expression decreased in a marginal manner (Figure 3e). Therefore, we concluded that CDC27 influences the cell cycle transition by mainly regulating the expression of p21. These results were confirmed using real-time PCR, and the data showed that p21 was negatively regulated by CDC27 at the mRNA level (Figure 3f). Moreover, the indicated stable cell lines were transfected with luciferase reporter plasmids driven by the p21 promoter. The results demonstrated that CDC27 inhibited p21 promoter activity (Figure 3g). Therefore, we concluded that the alteration of p21 expression can be at least in part attributed to the regulation of CDC27 at transcription level.

### CDC27 mediates p21-dependent cell cycle arrest by modulating ID1 expression

**Figure 4:**
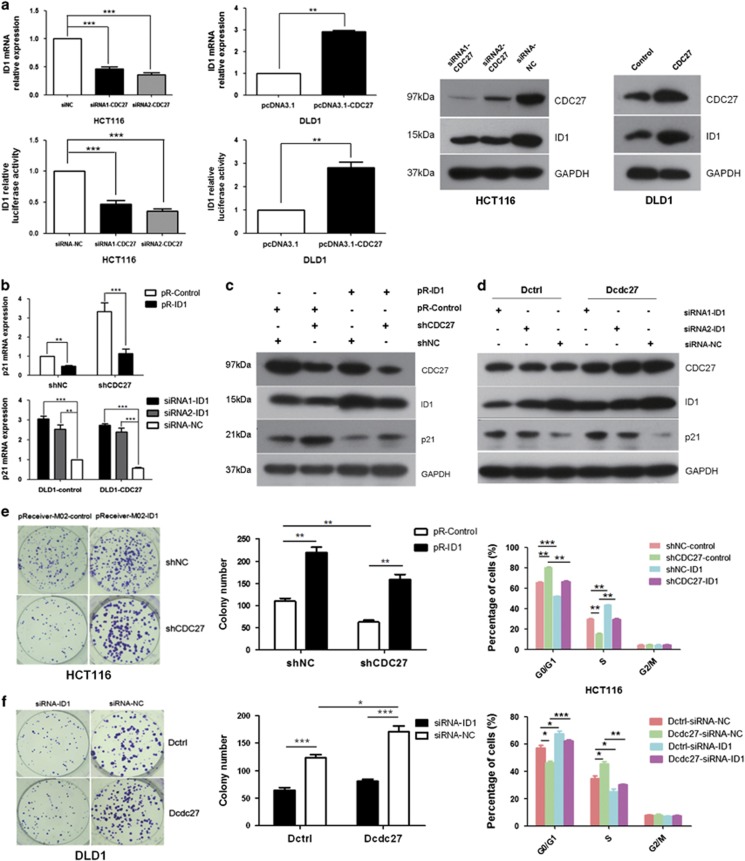
CDC27 regulates p21 expression through modulating ID1 expression. (**a**) Real-time PCR, luciferase reporter assays (left) and western blotting (right) were performed to detect ID1 expression in the indicated cells with CDC27 was transiently overexpressed or suppressed. Mean±S.D. of triplicate samples are shown. **P<0.01, ***P<0.001 using Student's *t*-test. (**b**–**d**) Cells were harvested at 4 h after synchronization by serum starvation for p21 mRNA and protein expression level detection. Mean±S.D. of triplicate samples are shown. **P<0.01, ***P<0.001, Student's *t*-test. (**e** and **f**) ID1 was transiently overexpressed or suppressed in the indicated stable cell lines. Representative images of colony formation rescue assays and quantification were presented. Cells were subjected to cell cycle distribution analysis by flow cytometry. Mean±S.D. of triplicate samples are shown. *P<0.05, **P<0.01, ***P<0.001, Student's *t*-test

ID1 is a key molecular regulator upstream of p21, and we wondered if ID1 participate in the regulation of p21 expression by CDC27. To test the hypothesis, we evaluated whether the expression of ID1 can be influenced by CDC27 using real-time PCR, luciferase reporter assays, and western blotting. Results indicated that ID1 expression was dramatically decreased upon transiently interference of CDC27 in HCT116 cells, whereas increased upon CDC27 overexpression in DLD1 cells (Figure 4a). Next, we transfected exogenous ID1 expression vectors (pR-ID1) or empty vector (pR-Control) into shCDC27 or shNC stable HCT116 cell lines, and simultaneously siRNAs of ID1 (siRNA1-ID1, siRNA2-ID1) or negative control sequence (siRNA-NC) were transfected into the Dcdc27 or Dctrl stable DLD1 cell lines. Alterations of p21 expression were detected by real-time PCR and western blotting. The results showed that overexpression of exogenous ID1 could significantly reduce p21 accumulation in HCT116-shCDC27 cell lines. Similarly, the p21 expression levels in Dcdc27 cells were increased after transient interference of ID1 (Figures 4b–d). Therefore, we concluded that CDC27 affects p21 expression in an ID1-mediated manner.

### Exogenous ID1 expression can reverse the inhibition of proliferation induced by CDC27 downregulation

Given the results obtained thus far, we next sought to determine whether ID1 affects the proliferation of CRC cells induced by CDC27. As shown in Figure 4e, the exogenous expression of ID1 reversed the inhibition of proliferation and suppressed the G1/S transition arrest caused by CDC27 downregulation in HCT116 stable cell lines. Moreover, transient transfection of ID1 siRNA suppressed cell proliferation and inhibited the G1/S transition caused by CDC27 in DLD1 stable cell lines (Figure 4f). Therefore, ID1 has a crucial role in CDC27-regulated CRC cell proliferation.

### CDC27 promotes tumor growth in a xenograft mouse model

**Figure 5:**
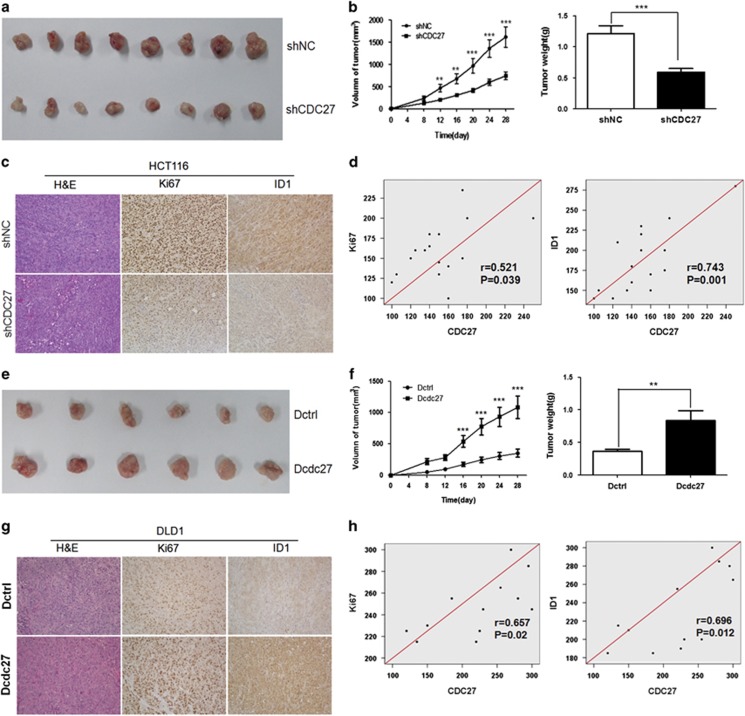
CDC27 promotes tumor growth *in vivo*. (**a** and **e**) The xenografts tumors were collected at 28 days after injection. (**b** and **f**) Data points are presented as the mean±S.D. tumor volume and the weight of the tumors were analyzed. **P<0.01, ***P<0.001. (**c** and **g**) The tumor sections were under H&E staining and immunohistochemstry staining using antibody against Ki67 and ID1 (× 200 magnification). (**d** and **h**) Correlations between selected molecular expression were analyzed by Pearson's test in mice tumor tissues

Next, we established a BALB/c nude mouse xenograft model using the stable HCT116 cell lines and DLD1 cell lines. Cells were injected subcutaneously into the flanks of male nude mice. Tumor size was measured every 4 days, and the tumor volume was calculated. Four weeks later, the mice were killed, and the tumors were collected. Results indicated that knockdown of CDC27 in HCT116 stable cell lines led to a significant decrease in tumor weight and volume compared with the control group (*n*=8, Figures 5a and b), whereas mice injected with Dcdc27 cells exhibited greater tumor growth capacity compared with the control group (*n*=6, Figures 5e and f). Furthermore, we used immunohistochemistry to evaluate the expression of ID1 and the nuclear cell proliferation marker Ki67 in the excised mouse tumors. As shown in Figure 5c, ID1 was stained in both the nuclear and cytoplasm. The tumors from the HCT116-shCDC27 group exhibited weaker Ki67 and ID1 staining intensity, whereas the tumors from the Dcdc27 group exhibited stronger Ki67 and ID1 staining intensity compared with the control group (Figure 5g). Moreover, the expression of both ID1 and Ki67 was positively correlated with CDC27 expression (Figures 5d and h). These results suggest that CDC27 contributes to tumor proliferation in CRC.

## Discussion

In the present study, our results showed that CDC27 has a key role in CRC proliferation through regulating p21 expression and consequently control G1/S phase transition. CDC27 may serve as an independent prognostic factor for CRC patients.

A major driver of cellular transformation is the loss of proper control of the mammalian cell cycle.^31^ Disturbance of this balance by disrupting the program that regulates cell cycle entry can result in the transformation of normal cells into tumor cells.^32, 33, 34^ As a key regulator of cell cycle progression at G1/S transition, p21 has a critical role in tumorigenesis.^34, 35, 36, 37^ Our study demonstrated that downregulation of CDC27 can lead to G1/S transition arrest via the accumulation of p21 (Figures 3b and d). We also performed apoptosis assays, although we did not find evidence that CDC27 has a key role in apoptosis (data not shown). In CRC, ID1 is also an important marker for tumor progression.^38^ Intriguingly, we observed CDC27 positively regulated ID1 expression in DLD1 and HCT116 cell lines (Figure 4a). To further investigate the relationship between CDC27 and ID1 in CRC, we detect the expression of CDC27 and ID1 both in CRC cell lines and 12 pairs of patient tissues. The results indicated the expression of CDC27 and ID1 is significantly correlated in the 12 pairs of patient tissues (Supplementary Figure 2A), despite that there was no correlation in CRC cell lines (Supplementary Figure 2B). Given that ID1 acts as a classical upstream regulator of p21,^30^ we next explored whether ID1 took part in p21 accumulation caused by CDC27 downregulation. Consistent with our assumption, exogenous overexpression of ID1 rendered cells' refractory to p21 accumulation (Figure 4c), and abolished the inhibition of proliferation caused by CDC27 downregulation (Figure 4e).

Dysregulation of APC/C has been shown to contribute to tumorigenesis.^39, 40^ APC/C is not only a complicated, large-size complex involving in various signaling pathways and cell biological process, but also works dependently on its subunits interaction and numerous substrates degradation in different phases of cell cycle, thereby any subunit or co-activator alteration may affect or even abolish its function and lead to cascade of response with a wide range of biological effects.^9, 10^ Several studies have shown that the two co-activators CDC20 and CDH1 act, respectively, as an oncogene and a tumor suppressor in cancer. However, few studies paid attention to the independent roles of its subunits in cancer. CDC27 is a core subunit of APC/C, but the function of CDC27 in cancer remains unclear. Regardless of APC function alteration, our study emphasized the role of CDC27 in CRC. To the best of our knowledge, our study is the first to explore the function of CDC27 in cancer, and our findings provide a novel perspective for further study of the functions of APC/C subunits.

Furthermore, it is worth mentioning that increasing evidence indicated that cancer stem-like cells (CSCs) are a rare subpopulation of cancer cells capable of propagating the disease and causing cancer recurrence, thus searching for the potential targeting of CSCs is a challenging task.^41, 42^ Previous studies have reported that p21 is linked to maintenance of self-renewal capacity in stem cells,^43, 44^ for that low level or absence of p21 expression leads to more efficient and faster reprogramming in mouse and human cells.^45, 46^ In addition, ID1 can also participates in self-renewal capacity in murine cortical neural stem cells, and studies in a murine model of hematopoiesis revealed that Id1^−/−^ whole-bone marrow displayed impaired self-renewal capacity relative to wild-type controls.^47^ Moreover, previous studies have demonstrated that ID1 participates in maintaining self-renewal through repressive effects on expression of p21 in hematopoietic stem cells and in bone marrow for the generation of endothelial progenitor cells.^48, 49^ In our study, we demonstrated that CDC27 regulated p21 expression through modulating ID1 expression. Therefore, we are curious about if CDC27 was involved in CSC. We have examined the expression of two classical colorectal epithelial stemness markers CD44 and CD133 using the HCT116 (shCDC27, shNC) and DLD1 (Dcdc27, Dctrl) stable cell lines. Interestingly, the expression of CD44 dramatically decreased in the HCT116-shCDC27 cell lines, and increased in the Dcdc27 cell lines compared with the control group (Supplementary Figure 3), whereas there was no difference in the expression of CD133. The findings above indicate there is a possibility that CDC27 is involved in CSC, and becomes a potential target of CSCs for monitoring the progress of cancer therapy and for evaluating new therapeutic approaches.

However, there are still many topics that remain to be addressed. First, recent studies have demonstrated that *CDC27* mutations are involved in various cancers; thus, we sought to investigate the presence of important disease-associated alleles, although we have not yet identified key mutations that can significantly affect tumor proliferation or progression. Second, ID1 is overexpressed in a variety of solid tumors,^50, 51, 52, 53^ and ID1 upregulation correlates with both poor prognosis and chemoresistance.^54, 55, 56, 57^ Therefore, we strongly suspect that CDC27 may also have a role in other cancers. Considering p21 is well positioned to function as both a sensor and an effector of multiple anti-proliferative signals.^58^ ID1 is not the unique mediator in CDC27-p21 regulation, and additional mechanisms may involve in p21 regulation caused by CDC27. In addition, for the reason that CDC27 expression is significantly associated with distant metastasis in CRC (Table 1), we have carried out experiments to explore the role of CDC27 in metastasis and invasiveness in CRC. Therefore, whether CDC27 can promote metastasis via modulating certain potential downstream molecular in CRC, and whether there is a correlation between CDC27 and its downstream molecular in clinical samples, remain to be determined in future studies.

In conclusion, our study is the first to demonstrate that CDC27 is associated with tumor proliferation and progression in CRC. These results suggest that CDC27 may serve as a prognostic biomarker and therapeutic target for CRC treatment.

## Materials and Methods

### Cell lines and cell culture

All cell lines used were purchased from the American Type Culture Collection (ATCC, Manassas, VA, USA), authentication by short tandem repeat profiling/karyotyping/isoenzyme analysis. Cell culture methods were listed in Supplementary Methods and Materials.

### Patient tissue specimens and clinicopathological characteristics

Patient tissues used in our study were collected from 166 patients histologically and clinically diagnosed with CRC between 1999 and 2005 at the Sun Yat-sen University Cancer Center (Guangzhou, China). The detailed selection criterion was described in the Supplementary Methods and Materials.

### Immunohistochemistry

The detailed method has been described in previous study.^59^ Primary antibodies used were as follow: CDC27 (Santa Cruz, CA, USA, sc-9972, 1 : 50), Ki67 (Santa, sc-15402, 1 : 100), and ID1 (Santa, sc-488, 1 : 100). The expression of CDC27 was evaluated using H-scores. The final H score was obtained according to the intensity and proportion of the area stained. CDC27 expression intensity was scored as negative=0, weak=1, moderate=2, or strong=3. The final H score was calculated by multiplying the intensity score by the percentage of the staining area. Receiver operating characteristic curve analysis was used to determine a cutoff value for CDC27 high expression or low expression. The sensitivity and specificity for the outcome under study was plotted, thus generating a receiver operating characteristic. The score that was closest to the point with both maximum sensitivity and specificity were selected as the cutoff value.

### RNA extraction and real-time PCR

Total RNA from colorectal cells was extracted using Trizol (Invitrogen, Carlsbad, CA, USA) reagent according to the manufacturer's instructions, and then cDNA was synthesized using the GoScript Reverse Transcription System (Promega, Madison, WI, USA). Real-time PCR was performed using a SYBR Green PCR Kit (Invitrogen). GAPDH was used as an endogenous control to normalize the amount of target gene mRNA. The primer sequences were shown in Supplementary Table 1.

### Stable cell line construction

CDC27-overexpressing or control vectors were transfected into DLD1 cells using Lipo2000. Cells were harvested at 48 h after transfection and were incubated with G418 for 12 days, with the medium refreshed every 3 days. For persistent suppression of CDC27 expression, short hairpin RNA oligonucleotides were cloned into the pSuper-retro-puro vector to knockdown CDC27 endogenous expression. Retrovirus vector pSuper-retro-puro-short hairpin RNA or pSuper-retro-puro empty vector and PIK packaging plasmids were cotransfected into 293T cells, and virus supernatant was harvested 48 h after transfection. Retrovirus supernatant was used to infect HCT116 cells, and puromycin was added 48 h after infection to select stable cell lines for 7 days, with the medium refreshed every 3 days.

### Western blotting

Total protein was extracted from cells using cell lysis buffer containing protease inhibitor (Sigma-Aldrich, St. Louis, MO, USA, P8340). Western blotting was performed as previously described.^59^ Primary antibodies used were summarized in Supplementary Methods and Materials.

### Colony formation and soft agar assays

For colony formation assays, HCT116 and SW480 cells were transfected with siRNAs of CDC27 or negative control siRNA, and DLD1 cells were transfected with CDC27-carring plasmids or empty vector. Cells were plated into six-well plates at 48 h after transfection. After incubating at 37 °C for 14 days, visible colonies were fixed and stained with 0.5% crystal violet in methanol. For the soft agar assay, the cells in single-cell suspension were plated in 0.3% agarose over a 0.6% agarose bottom layer in six-well plates and incubated for 14 days. The number of colonies containing more than 50 cells was counted.

### Cell cycle analysis

Cells were harvested, washed, and fixed in pre-cooled 75% ethanol mixed with PBS at −20 °C overnight. The next day, the cells were washed and resuspended in 500 *μ*l of pre-cooled PBS including RNase (Thermo Scientific, Lafayette, CO, USA) at a concentration of 10 *μ*g/ml, and the cells were incubated at 37 °C for 30 min before staining. A 30-*μ*l solution containing propidium iodide (Sigma) at a concentration of 1 mg/ml was added to the cells and incubated at 4 °C in the dark for at least 1 h. All cell samples were harvested and analyzed on a flow cytometer (Beckman Gallios, Beckman Gallios, Inc., Fullerton, CA, USA).

### Luciferase reporter assays

The luciferase reporter assays were performed as previously described.^60^

### Tumor formation assays in nude mice

Animal experiments were approved by the Sun Yat-Sen University Cancer Center Institutional Animal Care and Usage Committee. Male BALB/c nude mice (4–5 weeks old, 15–18 g) were purchased from the Slaccas Experimental Animal Center (Shanghai, China). Mice were injected with HCT116 or DLD1 stable cell lines (*n*=8 and *n*=6, respectively). 2 × 10^6^ cells were injected subcutaneously into each nude mouse. The tumor volume was calculated using the following formula: *V*=(width^2^ × length)/2. After 28 days, the mice were euthanized, and the primary tumors were collected and weighed.

### Statistical analysis

All statistical analyses were completed using the SPSS version 16.0 statistical software package (SPSS Inc., Chicago, IL, USA). Distributed data are expressed as mean±S.D. Student's *t*-test and two-tailed *χ*^2^ test were used. Pearson's test was applied for correlation analysis. A *P* value of less than 0.05 was considered statistically significant.

## Figures and Tables

**Table 1:** Clinicopathological findings and correlation with CDC27 expression

**Variables**	***N*** **(%)**	**CDC27-low (%)**	**CDC27-high (%)**	***P*** **value**
Total cases	166	75 (45.2)	91 (54.8)	
				
*Age (years)*				
<60	116 (69.9)	53 (31.9)	63 (38.0)	0.867
≥60	50 (30.1)	22 (13.4)	28 (16.7)	
				
*Gender*				
Male	89 (53.6)	37 (22.3)	52 (31.3)	0.35
Female	77 (46.4)	38 (22.9)	39 (23.5)	
				
*Tumor location*				
Colon	88 (53.0)	38 (22.9)	50 (30.1)	0.64
Rectum	78 (47.0)	37 (22.3)	41 (24.7)	
				
*Tumor size (cm)*				
<5	60 (36.1)	34 (20.5)	26 (15.7)	0.035^a^
≥5	106 (63.9)	41 (24.7)	65 (39.2)	
				
*Tumor invasive depth*^b^				
T1–T2	39 (23.5)	19 (11.4)	20 (12.0)	0.713
T3–T4	127 (76.5)	56 (33.7)	71 (42.8)	
				
*AJCC/TNM stage*				
I-II	75 (45.2)	42 (25.3)	33 (19.9)	0.013^a^
III-IV	91 (54.8)	33 (19.9)	58 (34.9)	
				
*Lymph node status*				
<1	91 (54.8)	47 (28.3)	44 (26.5)	0.085
≥1	75 (45.2)	28 (16.9)	47 (28.3)	
				
*Distant metastasis*				
No metastasis	126 (75.9)	63 (37.95)	63 (37.95)	0.03^a^
Metastasis	40 (24.1)	12 (7.2)	28 (16.9)	
				
*Preoperative CEA (ng/ml)*				
<5	81 (48.8)	38 (22.9)	43 (25.9)	0.755
≥5	85 (51.2)	37 (22.3)	48 (28.9)	

Abbreviation: CEA, carcino-embryonic antigen.

Note: The numbers in parentheses indicate the percentages of tumors with a specific clinical or pathologic feature for a given CDC27 subtype.

a Statistically significant.

b According to the 7th Edition of the AJCC Cancer Staging Manual.

**Table 2:** Multivariate analysis for PFS and OS

**Variable**	**PFS, HR (95% CI)**	***P*** **value**	**OS, HR (95% CI)**	***P*** **value**
Age (years, <60 *versus* ≥60)	1.11 (0.63–1.97)	0.70	1.21 (0.72–2.03)	0.48
Gender (male *versus* female)	1.13 (0.68–1.88)	0.64	1.25 (0.78–2.00)	0.35
Tumor location (colon *versus* rectum)	1.18 (0.70–1.99)	0.53	1.49 (0.93–2.41)	0.10
Tumor size (cm, <5 *versus* ≥5)	1.07 (0.56–2.05)	0.84	1.17 (0.66–2.08)	0.59
Tumor invasive depth (T1–2 *versus* T3–4)	0.84 (0.32–2.2)	0.72	0.88 (0.37–2.06)	0.76
TNM stage (I-II *versus* III-IV)	10.65 (5.2–21.7)	<0.001^a^	8.46 (4.69–15.2)	<0.001^a^
Lymph node status (<1 *versus* ≥1)	2.21 (1.17–4.19)	0.02^a^	2.55 (1.41–4.64)	0.002^a^
Distant metastasis (no *versus* yes)	7.4 (4.02–13.65)	<0.001^a^	6.53 (3.74–11.48)	<0.001^a^
CDC27 (low *versus* high)	1.78 (1.03–3.07)	0.04^a^	1.79 (1.09–2.94)	0.02^a^

Abbreviations: PFS, progression-free survival; OS, overall survival; HR, hazard ratio; CI, confidence interval.

a Statistically significant.

## Abbreviations

CRC: colorectal cancer
CDC27: cell division cycle 27
APC/C: anaphase-promoting complex/cyclosome
ID1: inhibitor of DNA binding 1
CDC20: cell division cycle 20
PFS: progression-free survival
OS: overall survival
siRNA: small interfering RNA
CSC: cancer stem-like cell
