# Supplementary material for: Downregulation of CDC27 inhibits the proliferation of colorectal cancer cells via the accumulation of p21Cip1/Waf1
Source: Cell Death Dis. 2016 Jan 28;7(1):e2074–. doi: 10.1038/cddis.2015.402 (PMC4816181; doi:10.1038/cddis.2015.402)
Supplement: Supplementary Information [file cddis2015402x1.docx]

**Supplementary methods and materials**

**Cell lines and cell culture**

FHC cells were cultured in Dulbecco’s modified Eagle’s medium (DMEM):F12 medium (containing 10 ng/mL cholera toxin, 0.005 mg/ mL insulin, 0.005 mg/ mL transferrin, and 100 ng/ mL hydrocortisone). HCT116 and COLO205 cells were cultured in DMEM with 10% fetal bovine serum (FBS, PAA). DLD-1 cells were maintained in 1640 medium containing 10% FBS. SW480 cells were cultured in L15 medium with 10% fetal bovine serum. All cell lines were tested for mycoplasma contamination (New MycoProbe Mycoplasma Detection Kit, R&D Systems) at least every month and were passaged for a maximum of 2 months, after which new seed stocks were thawed for experimental use.

**Patient tissue specimens and clinicopathological characteristics**

The patients were followed-up once every 3 months during the first 2 years, once every 6 months during the third and fourth year, and once a year from the fifth year postoperatively. The patients who did not have follow-up information were excluded from this study. The collected tissue specimens conformed to the criteria that they contained matched tumors (percentage of tumor cells > 70%) and corresponding normal mucosal tissue (>5 cm laterally from the edge of the tumor region). Patients who had a single primary lesion and no neoadjuvant chemotherapy were included. The use of clinical specimens for research purposes was approved by the Institutional Research Ethics Committee, and the patients were informed prior to the use of clinical material.

**Plasmid vector construction and cell transfection**

Full-length CDC27 cDNA was generated by PCR amplification and cloned into pcDNA3.1 (Invitrogen). ID1-carrying plasmids (EX-F0699-M02) and empty control plasmids were purchased from FulenGen Company (Shanghai, China). DLD1 and HCT116 cells were seeded in six-well plates and then transfected with plasmid or the corresponding empty control vector plasmid using Lipofectamine 2000 (Lipo2000) transfection reagent (Invitrogen). Cells were harvested at indicated time point for Western blotting and Real-time PCR analysis. The siRNA (GenePharma Company, Shanghai, China) sequences were as follows: CDC27 #1, 5’-GGAAAUAGCCGAGAGGUAA-3’, and #2, 5’-GGUCCACAAACAAGUACAA-3’; ID1: #1, 5’-AUAUUACAAUGAUCACCGACUGAAA-3’, and #2, 5’-GGAAUUACGUGCUCUGUGGGUCUCC-3’. A negative control sequence 5’-CGUACGCGGAAUACUUCGA-3’ was also used. Growing cells were seeded in a six-well plate, and siRNAs were added 24h later at a concentration of 100 nM using 5 µL of Lipo2000 reagent.

**Antibodies**

The primary antibodies used for western blotting included CDC27 (Santa Cruz sc-9972; 1:200), ID1 (Santa Cruz, sc-488; 1:200), GAPDH (Santa Cruz; 1:2,000), p21 (Santa Cruz, sc-6246; 1:200), p27 (Santa Cruz, sc-528; 1:200), CDK2 (Santa Cruz, sc-163; 1:200), CD44 (Cell signaling technology, #3570; 1:1000), and CD133 (Proteintech, 18470-1-AP; 1:1000)
