# Supplementary material for: Downregulation of CDC27 inhibits the proliferation of colorectal cancer cells via the accumulation of p21Cip1/Waf1
Source: Cell Death Dis. 2016 Jan 28;7(1):e2074–. doi: 10.1038/cddis.2015.402 (PMC4816181; doi:10.1038/cddis.2015.402)
Supplement: Supplementary Table 1 [file cddis2015402x2.docx]

**Supplementary Table 1**

**Primer sequences for real-time PCR**

| **Gene** | **Sense primer** | **Antisense primer** |
| --- | --- | --- |
| CDC27 | ACTAAACCACTATGCTTACCGAGATG | CACTCCACCAGATAAGATTTGTTCC |
| p21 | TGTCCGTCAGAACCCATGC | AAAGTCGAAGTTCCATCGCTC |
| ID1 | CGTGCTGCTCTACGACATGA | GCTCCAACTGAAGGTCCCTG |
