# Supplementary material for: Downregulation of CDC27 inhibits the proliferation of colorectal cancer cells via the accumulation of p21Cip1/Waf1
Source: Cell Death Dis. 2016 Jan 28;7(1):e2074–. doi: 10.1038/cddis.2015.402 (PMC4816181; doi:10.1038/cddis.2015.402)
Supplement: Supplementary Figures [file cddis2015402x3.docx]

**Figure S1. Knockdown of CDC27 inhibits proliferation in SW480 cells.** Representative images show the colony formation ability of SW480 cells with CDC27 transiently suppressed. The number of the colonies was quantified. Error bars, mean ± SD; ***, *p*<0.001 using Student’s *t*-test.

**Figure S2: Analysis of the correlation between CDC27 and ID1 expression. (A)** Representative images of CDC27 or ID1 expression in CRC tissues by immunohistochemistry (200 X magnification). Correlations were analyzed between ID1 and CDC27 expression by Pearson’s test in 12 patient tissues. **(B)** ID1 protein expression was detected by western blot analysis in a normal colon epithelial cell line (FHC) and 7 CRC cell lines. GAPDH was used as a reference control.

**Figure S3: CDC27 promotes the expression of CD44.** Western blotting analysis was used to detect alterations in CD44, CD133 expression in the indicated cell lines. GAPDH was used as a reference control.
