# Supplementary figures and images for: Downregulation of CDC27 inhibits the proliferation of colorectal cancer cells via the accumulation of p21Cip1/Waf1
Source: Cell Death Dis. 2016 Jan 28;7(1):e2074–. doi: 10.1038/cddis.2015.402 (PMC4816181; doi:10.1038/cddis.2015.402)

# Figure S1

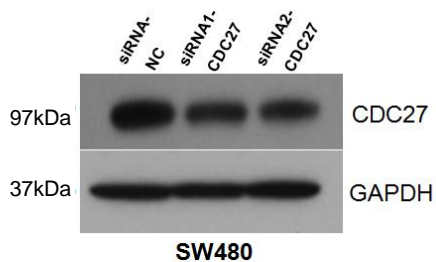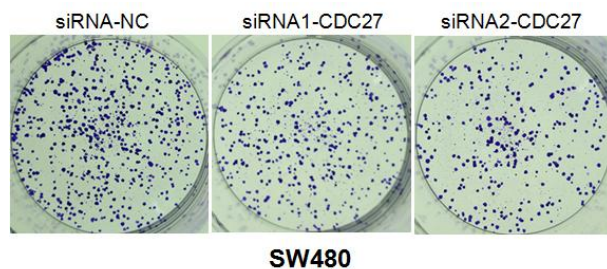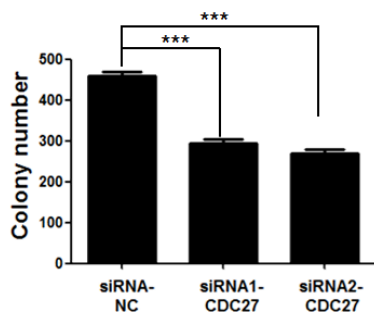

Supplement: Supplementary Figure S1 [file cddis2015402x4.pdf]

# Figure S2

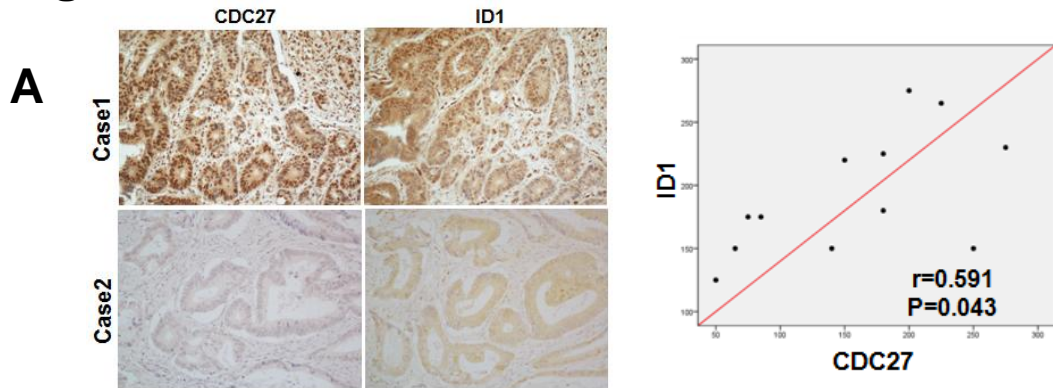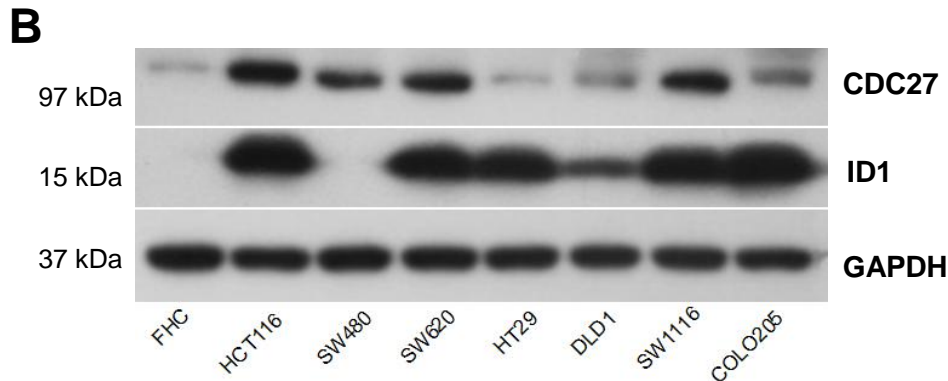

Supplement: Supplementary Figure S2 [file cddis2015402x5.pdf]

**Figur S3**

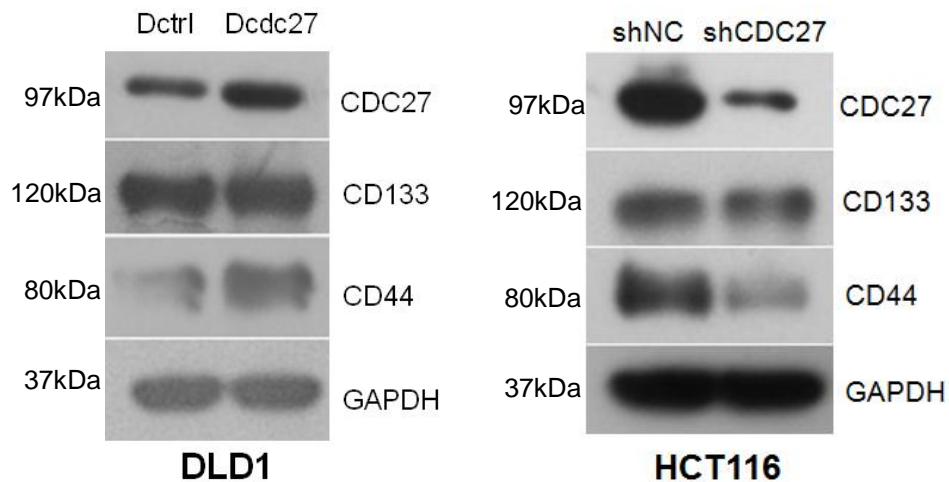

Supplement: Supplementary Figure S3 [file cddis2015402x6.pdf]
